# Cellular senescence in cancer immunology and potential therapeutic strategy

**DOI:** 10.3389/fonc.2026.1737652

**Published:** 2026-04-01

**Authors:** Xiao-Dan Qian, Li-De Tao, Li-Hong Zhang, An-Lai Ji, Lei Wang, Muhammad Mudasar Iqbal, Yi Luo

**Affiliations:** 1 Department of Paediatrics, Northern Jiangsu People's Hospital, Yangzhou, Jiangsu, China; 2 Department of Hepatobiliary Surgery, The Affiliated Hospital of Yangzhou University, Yangzhou, Jiangsu, China

**Keywords:** cellular senescence, senescence-associated secretory phenotype, immunosuppressive, immune evasion, immunotherapy

## Abstract

Cellular senescence represents a state of stable, often irreversible cell cycle arrest. Unlike apoptosis, senescent cells (SCs) remain metabolically active and engage in robust secretory activity, most notably through the senescence-associated secretory phenotype (SASP). The SASP exerts profound and context-dependent effects on tumor initiation and progression. This review analyzes the dual role of senescent cells in tumor immunity. On one hand, they can exhibit anti-tumorigenic effects through SASP-mediated enhancement of immune surveillance and their inherent high immunogenicity. On the other hand, they can promote tumorigenesis by fostering an immunosuppressive microenvironment, polarizing immune cells via the SASP, and upregulating senescence-associated immune checkpoints (SAICs) to facilitate immune escape. These dual characteristics inform promising therapeutic strategies: first, inducing senescence in tumor cells, and second, selectively eliminating the resulting senescent populations. Notably, systemic senescence induction can cause off-target effects in healthy tissues, underscoring the need for targeted delivery systems. In conclusion, we highlight emerging senescence-targeted immunotherapies as a next-generation approach to strategically harness senescence for cancer control.

## 1 Introduction

**Figure 1:**
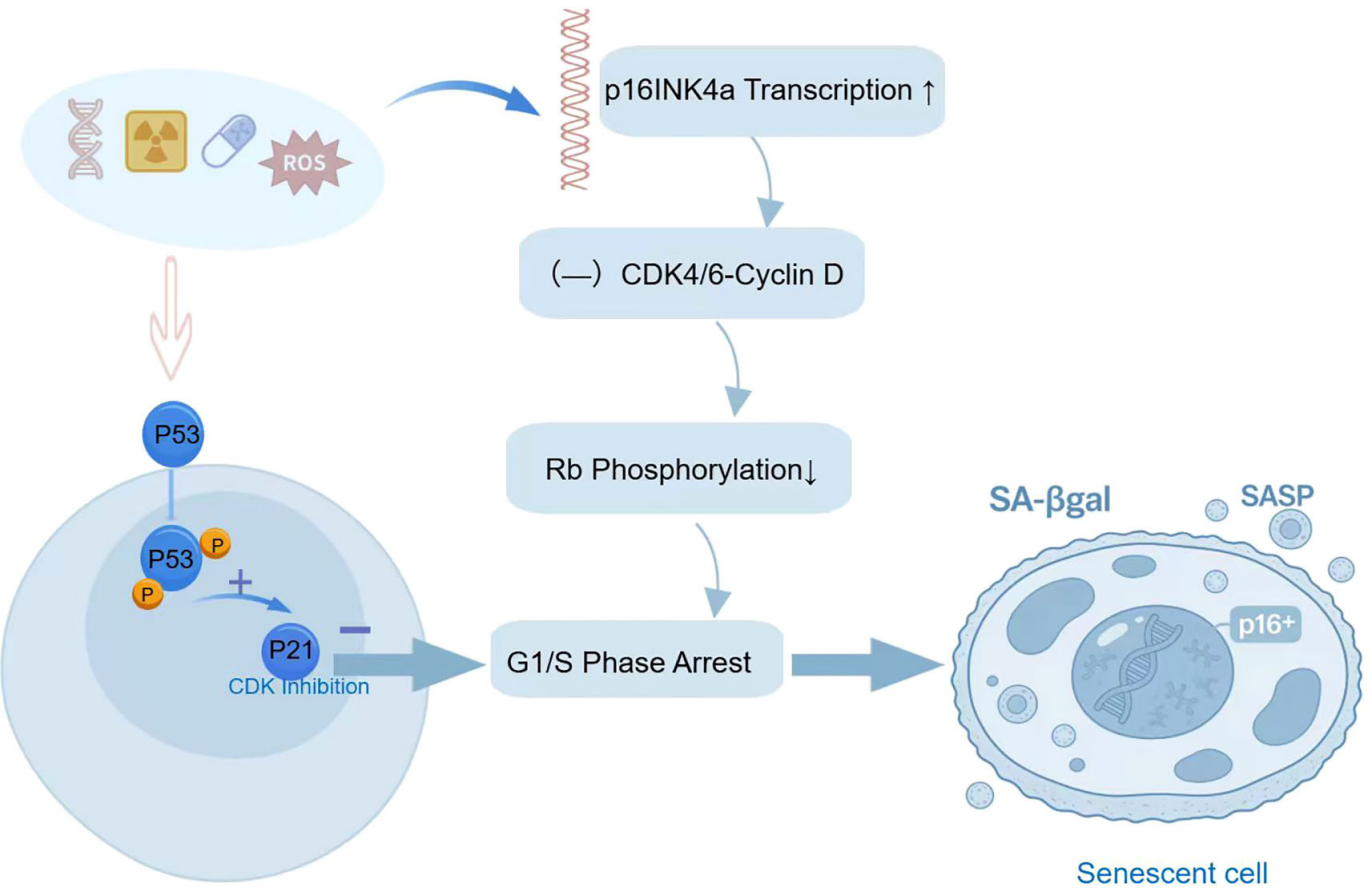
p53-p21 pathways: In response to severe DNA damage like double-strand breaks, the ATM/ATR kinases phosphorylate and activate p53. The stabilized p53 protein enters the nucleus and acts as a transcription factor to induce the expression of p21 (encoded by CDKN1A). By inhibiting CDK2/cyclin E and CDK4/6/cyclin D complexes, p21 prevents Rb phosphorylation. Consequently, cells are arrested at the G1/S checkpoint, resulting in irreversible growth arrest characteristic of senescence, p16-Rb pathways: Chronic stress, including telomere dysfunction, oxidative damage, and persistent oncogenic signaling, induces the transcriptional upregulation of the p16INK4a gene. The p16 protein specifically binds to and inhibits the kinase activity of CDK4/6-cyclin D complexes, thereby blocking the phosphorylation of the retinoblastoma (Rb) protein. This maintains Rb in its active, hypo-phosphorylated state, which in turn irreversibly suppresses E2F-mediated cell cycle progression. Consequently, cells enter a state of permanent growth arrest, a defining feature of cellular senescence.

Cellular senescence refers to the irreversible growth arrest state that cells enter as their proliferative capacity declines over time or due to damage (including chemotherapy, ionizing radiation, oncogene activation, oxidative stress and so on) during the course of their life activities, which involving a programmed stress response regulated by defined molecular pathways, such as p53-p21, p16-Rb pathways (**Figure 1**) (1, 2). Senescent cells (SCs) still alive and have metabolic activity unlike apoptotic cells. This phenomenon was firstly identified in human diploid fibroblasts by Hayflick and Moorhead in 1961, which originally described as an irreversible cell-cycle arrest mechanism that suppresses tumorigenesis (3). Cellular senescence primarily falls into three categories: replicative senescence, which results from telomere shortening; developmentally programmed senescence, a vital process for proper embryogenesis; and stress-induced premature senescence(SIPS), triggered by various stimuli (4). SIPS is an early aging state induced by external and internal stress factors. Its characteristics are similar to replicative senescence, but it occurs before telomere length is exhausted. It usually means a “emergency brake” mechanism of the body in response to major injuries or stress, used to prevent damaged cells from continuing to divide, thereby preventing potential tumor occurrence (5). This is a direct bridge for tumor treatment (such as chemotherapy, radiotherapy, targeted drugs) to induce senescence (TIS). However, its long-term existence can lead to the decline of tissue function and the promotion of tumor growth (5, 6). Rather than becoming inert, senescent cells activate a pro-inflammatory secretome known as the senescence-associated secretory phenotype (SASP). This phenotype is characterized by the sustained release of a complex mixture of signaling molecules—including cytokines, chemokines, growth factors, and proteases. Ultimately, the SASP acts as a potent molecular beacon, broadcasting the senescent state to the entire tissue microenvironment (7). The discovery of the SASP marks a paradigm shift in our understanding of cellular senescence, revealing that senescent cells influence the tissue microenvironment through non-cell-autonomous mechanisms rather than purely cell-intrinsic effects (8, 9). While cellular senescence serves as a fundamental anti-proliferative mechanism across physiological and pathological contexts, its roles are profoundly context-dependent. The senescence program engaged in wound healing acts as a transient, pro-regenerative force, distinct from its chronic, detrimental role in organismal aging. In cancer, senescence assumes a particularly complex and dualistic identity: it can function as a potent tumor-suppressive barrier or, paradoxically, as a catalyst for tumor progression via its secretome (10).This raises a central, unresolved question in oncology: How do SCs, particularly within the tumor ecosystem, interact with the immune system, and how does this crosstalk ultimately dictate tumor fate—toward suppression or progression?

The immune system is not only a guardian against pathogens but also an internal tumor surveillance mechanism. Through the process of immunoediting, tumor cells that survive the elimination phase can actively sculpt the tumor immune microenvironment (TIME) into an immunosuppressive niche, effectively combating host immunity. The balance between pro- and anti-tumor inflammatory mediators may determine tumor progression (11).Within the TIME, the SCs are functionally heterogeneous. It encompasses therapy-induced senescent tumor cells alongside senescent stromal cells (e.g. cancer-associated fibroblasts and endothelial cells), the latter often entering senescence in response to oncogenic signals or therapy. Through their integrated secretome, this collective senescent niche actively orchestrates the local immune response (12). Under normal physiological conditions, the SCs are effectively eliminated through a well-coordinated immune response in immunocompetent hosts. This process involves multiple components of the innate and adaptive immune systems, including macrophages, neutrophils, natural killer (NK) cells, and CD4^+^ T cells. Moreover, SASP supports this clearance by releasing chemokines and cytokines that act as attractants, specifically recruiting NK cells and other immune effectors to the sites of SCs accumulation (13). However, in pathological contexts such as cancer, the SCs can evade this immune surveillance. They primarily achieve escape by upregulating immune checkpoint molecules (e.g. PD-L1) and secreting factors like matrix metalloproteinases (MMPs). These mechanisms collectively disrupt normal clearance pathways and foster an immunosuppressive microenvironment, which further perpetuates their persistence (14). The SCs play the role of “both enemy and friend” in caner, and its’ ultimate effect is highly dependent on the efficiency of the immune system in recognizing and eliminating them. While recent reviews have comprehensively cataloged the dual roles of senescent cells in promoting both immune surveillance and evasion (15), this review aims to move beyond the simplistic ‘friend or foe’ dichotomy and provide an integrated framework that delineates how the balance between senescence-mediated immune activation and suppression is controlled. Focusing on this balance will illuminate novel strategies to therapeutically reprogram the senescent-immune dialogue for improved cancer outcomes.

## 2 SCs- The “Good Partner” for anti-tumor immunity (promote clearance)

### 2.1 Enhance immunogenicity and antigen presentation (“Eat me”)

The execution of immune surveillance critically depends on the specific recognition of the by immunocompetent effector cells. The SCs do not passively wait to be identified. Instead, they actively upregulate a series of “danger signal” molecules and secrete chemokines to transform themselves into high-priority targets of the immune system, thereby initiating the clearance process (16). Natural killer group 2D (NKG2D) is a stimulatory receptor predominantly expressed on the surface of natural killer (NK) cells and certain T cell subsets. The upregulation of its cognate ligands (NKG2DLs) is not merely a biomarker but a functional vulnerability shared by stressed cells, whether malignant or senescent. This has been robustly demonstrated not only in cultured SCs but also *in vivo*, such as in skin fibroblasts from aged individuals, positioning these ligands as key “stress-induced self” markers that flag senescent cells for immune recognition (17, 18). Ines Marin et al. showed that SCs significantly up-regulate the expression of major histocompatibility complex class I (MHC-I) molecules, while interferon (IFN) signaling, specifically type I, is a key mediator of MHC-I upregulation in SCs *in vitro*. Moreover, they can release more antigenic peptides, which can be efficiently captured by dendritic cells (DCs), thereby activating CD8-positive T lymphocytes (CD8^+^ T cells). This indicates that senescent tumor cells can be exploited to develop efficient and protective CD8-dependent antitumor immune responses (19). Hsuan-An Chen et al. established a genetically engineered “senescence-inducible” liver cancer model, demonstrating that, apart from sustained secretion of chemokines and cytokines to shape a pro-immunogenic microenvironment, senescence can also drive a major remodeling of the cell-surface proteome and signaling program, which fundamentally alter the way cells sense and respond to environmental signals through a hypersensitivity to microenvironmental type II IFN (IFNγ), robustly inducing the antigen-presenting mechanism, each process is required for their effective immune surveillance (20). Among them, MHC class I chain-related proteins A/B (MICA/B) are expressed only in human cell, which are considered a general feature of human senescent cells (21). These proteins are essential for NK cell-mediated immunosurveillance, enabling the recognition and elimination of senescent cells via the granule exocytosis pathway. *In vitro*, irradiation-induced senescence on primary fibroblasts leads to non-classical MHC class I MICA/MICB expression, allowing CD8+ T cell-mediated cytolysis effects (22).

### 2.2 Recruit immune cells into their microenvironment to activate immune surveillance (“Find me”)

SCs are not only static targets of immune attacks but also active signaling centers. Wen Xue et al. first demonstrated that the innate immune response eliminates the SCs induced in tumors via hepatocellular carcinoma mouse models (23).This is closely associated with SASP, which comprises chemokines and cytokines that bind to specific receptors on immune cells (24).By secreting a specific set of chemokines, they actively recruit and guide immune cell subpopulations, initiating the clearance process. The SASP of SCs contains a characteristic chemokine spectrum, each of which is responsible for attracting specific immune effectors. C-C Motif Chemokine Ligand 2 (CCL2), secreted by senescent hepatocytes, can bind to C-C Chemokine Receptor Type 2 (CCR2), which is expressed on the surface of monocytes/macrophages and neutrophilic granulocytes, and then mediate the immune clearance of senescent cells and inhibit tumorigenesis. This phenomenon, termed ‘senescence surveillance’ was described by Tobias Eggert et al. as an important tumor suppressive barrier in the body (9, 25). Additionally, researchers found the level of C-X-C motif chemokine ligand 10 (CXCL10) increased significantly in senescent hepatocyte model. They can specifically bind to the C-X-C Chemokine Receptor Type 3 (CXCR3) on NK cells, which can recruit and activate NK cells to enhance immune surveillance and eliminate these SCs, thereby suppressing hepatic fibrosis or hepatocellular carcinoma progression (26).

### 2.3 Immune cell-mediated clearance of SCs (“Kill me”)

The mechanism of senescent cell clearance mediated by immune cells can be divided into two categories: innate immune clearance and adaptive immune clearance. The function of this immune reaction appears to be the killing and eventual clearance of senescent cells, as well as the stimulation of a local immune response to eliminate oncogene-expressing cells, including those that have undergone oncogene-induced senescence and those oncogene-transformed cells that have bypassed or escaped senescence (27). In the immune system’s initial response to eliminate SCs, NK cells serve as essential “first responders”. Operating independently of prior antigen sensitization, they rapidly detect alarm signals released by SCs. Through direct cytotoxic mechanisms and the release of effector cytokines, NK cells efficiently orchestrate the early phases of senescent cell clearance. As previously noted, SCs characteristically upregulate the expression of ligands for the activating receptor NKG2D. Binding of the NKG2D receptor on NK cells to these cognate ligands delivers a potent activating signal. This signal directly orchestrates the cytoskeletal polarization of cytotoxic granules toward the immune synapse and triggers their subsequent exocytosis, a process that mediates target cell lysis via the release of pore-forming perforin and pro-apoptotic serine proteases, notably granzymes (28). Macrophages serve as another pivotal effector cells in the clearance of SCs, fulfilling roles that extend well beyond mere scavenger functions. Their phenotypic plasticity allows them to polarize into distinct functional subtypes in response to specific microenvironmental cues. Within this spectrum, classically activated M1-type macrophages are critically involved in both the phagocytic elimination of senescent cells and the subsequent initiation of adaptive immune responses (29). M1-type macrophages recognize specific “eat-me” signals displayed by SCs. Following phagocytosis, they orchestrate a sophisticated antigen processing and presentation cascade. Processed antigens derived from internalized SCs can be presented via two major pathways: (1) the major histocompatibility complex (MHC) class II pathway to activate CD4^+^ T helper cells, and (2) the MHC class I cross-presentation pathway to directly prime CD8^+^ cytotoxic T cells. This dual-antigen presentation capability bridges innate clearance with the activation of a broad, antigen-specific adaptive immune response (30). The enhanced immune activity of senescent cells is also related to their immune microenvironment. They can exert a powerful tumor suppressive function by remodeling the immune microenvironment, such as altering the composition of immune cells in the tumor microenvironment and reducing the number of immunosuppressive cells (31).In addition, emerging evidence suggests that the eukaryotic translation initiation factor 5A (eIF5A) plays an important role in cellular senescence, potentially by affecting the expression of SASP components and functions of the immune system (32). Researchers have established that SCs upregulate global protein synthesis relative to their proliferating counterparts, with the translation initiation factor eIF5A identified as a key node governing this biosynthetic shift. Furthermore, studies have delineated a p53-dependent transcriptional program in senescent cells that sustains the hypusination of eIF5A—an essential post-translational modification. This regulatory axis is critically required for the effective immune-mediated clearance of pre-malignant senescent cells, thereby linking cellular senescence, translational control, and tumor immunosurveillance (33).

The mechanisms described above collectively depict an ideal paradigm in which SCs function as endogenous immune “Good Partner”. By releasing danger signals, presenting antigens, and fostering a pro-inflammatory milieu, they effectively prime innate and adaptive immunity and undergo subsequent clearance, thereby executing their tumor-suppressive mandate. However, this sequence is not invariably executed to completion. When immune-mediated clearance fails and SCs persist and accumulate within tissues, their robust secretory capacity—evolutionarily intended to signal distress—transforms from a protective asset into a pathogenic liability. The persistent, dysregulated secretion of SASP factors, particularly a subset of key mediators, can subvert an initially acute, resolutive inflammatory response into a chronic pathological state that fosters immunosuppression, tissue dysfunction, and tumor progression (5, 10). In the following section, we will delineate the molecular mechanisms underlying this deleterious transition.

## 3 SCs — an “accomplice” to pro-tumor immunity

### 3.1 Create an immunosuppressive environment

Through the sustained secretion of the SASP, SCs establish a profoundly immunosuppressive microenvironment conducive to tumorigenesis. Key SASP factors—including IL-6, IL-1β, IL-8, TGF-β, and PGE_2_—collectively remodel the tumor microenvironment and directly inhibit the function of effector immune cells, thereby subverting immunosurveillance and impairing the clearance of malignant cells (34). Among these, IL-6 functions as a central node. It promotes tumor cell proliferation and invasion by activating the NF-κB and JAK2/STAT3 signaling pathways (35, 36). Furthermore, IL-6 facilitates the recruitment and activation of immunosuppressive myeloid-derived suppressor cells (MDSCs) and M2-type macrophages, which in turn suppress the cytotoxic activity of CD8^+^ T cells and NK cells, thereby attenuating anti-tumor immunity (37, 38). The net biological effect of IL-6, however, is context-dependent, influenced by the integration of specific signaling pathways, the broader microenvironmental milieu, and therapeutic interventions (39). Another critical axis is the CXCL12/CXCR4 signaling pathway, which can be activated by senescent tumor cells. In pancreatic cancer, CXCL12/CXCR4 engages downstream effectors such as FAK, ERK, and AKT to drive oncogenic signaling (40), inducing activation of mitogen-activated protein kinase (MAPK) in ovarian cancer cells enhancing cell survival and treatment resistance (41). Moreover, it can affect the differentiation and function of immune cells. The CXCL12-mediated effects on adaptive immunity are mainly detrimental and affect mostly T cells. For instance, it can upregulate immunosuppressive Forkhead box protein P3-positive regulatory T cells (FOXP3^+^ Tregs) and downregulate CD8+ T-cell content in basal-like breast cancer (42). In models of oncogenic-induced hepatocyte senescence, researchers showed that the growth of tumor cells is more aggressive in the presence of senescent cells compared with non-senescent cells, due to activation of the CCL2-CCR2 pathway, thereby promoting the accumulation of immunosuppressive myeloid cells, preventing natural killer (NK) cells from clearing the tumor cells (25).

### 3.2 Transform the polarization state of immune cells

Immune cells usually exhibit two polarization states: classically activated M1 cells implicated in pro-inflammatory and anti-tumorigenic effects, while M2 or M2-like cells, which are associated with resolution or smoldering chronic inflammation (43). SCs can transform the polarization state of immune cells through the secretion of SASP, thereby promoting tumor progression. Mara Mazzoni, et al. constructed an *in vitro* cell interaction model, simulating different stages of thyroid tumors, in which purified peripheral blood-derived human monocytes were exposed to them, found that senescent thyrocytes and thyroid tumor cells induced M2-like macrophage polarization of human monocytes via a PGE2-dependent mechanism (44, 45). The resulting M2-like macrophages displayed tumor-promoting activity, a conclusion also confirmed by the study of C Zhang, et al (46). In another *in vitro* study, researchers found that the secretome from senescent hepatoma cells induced macrophage differentiation predominantly with M2 markers, whereas that of non-senescent cells induced M1 phenotype, thereby modulating the microenvironment toward tumor promotion (47).

### 3.3 Express senescence associated immune checkpoints

Moreover, senescent cells can bypass immunosurveillance and promote immune escape through multiple mechanisms, involving the expression of secreted or cell surface bound molecules, collectively referred to as senescence associated immune checkpoints (SAICs), including programmed cell death 1 ligand 1 (PD-L1) and programmed cell death 1 ligand 2 (PD-L2) which bind to programmed cell death protein 1 (PD-1) on T cells, as well as non-classical major histocompatibility complex (MHC) (48). The interaction between PD-1 and PD-L1 negatively regulates adaptive immune response, mainly by inhibiting the activity of effector T cells while enhancing the function of immunosuppressive regulatory T cells (Tregs), and cancer cells can exploit the PD-1/PD-L1 axis to enable immune escape during cancer development and progression (49). Senescent cells also upregulate the immune checkpoint molecule PD-L1 through SASP secretion and activation of the JAK-STAT pathway, thereby driving immune cell inactivation and promoting immune escape (50). In the study of Yang Chen et al, boningmycin was used to induce senescence in human lung cancer cells and breast cancer cells (BON) and found the upregulation of PD-L1 in these cells, thereby confirming the above observations (51). Interestingly, PD-L2 can also weaken the anti-tumor immune response by binding to PD-1 and inhibiting the activity of T cells, thereby exerting a similar effect of PD-L1. In animal models, researchers showed that PD-L2 expression—either alone or co-expressed with PD-L1— in tumor cells significantly suppressed antitumor immune responses (52). Senescent cells are closely related to the upregulation of PD-L2, and play a key role in the immune escape of senescent cells in tumors. Selim Chaib and his colleagues performed an unbiased proteomic screen of plasma membrane proteins in senescent and non-senescent human melanoma cells, using doxorubicin or palbociclib to trigger therapy-induced senescence (TIS), and discovered that among the upregulated plasma membrane proteins in both TIS conditions was PD-L2 (53), which affected the response to chemotherapy. Similarly, changes in major histocompatibility complex (MHC) expression in senescent cells can lead to escape from recognition by the immune system. Researchers showed that senescent dermal fibroblasts express the non-classical MHC molecule HLA-E, which interacts with the inhibitory receptor NKG2A expressed by NK cells and highly differentiated CD8+ T cells, thereby suppressing the immune attack on senescent cells (54). In another *in vivo* senescence model, researchers innovatively revealed the metabolic landscape of the senescent tumor microenvironment, characterized by elevated adenosine levels, attributed to senescent tumor cell-induced CD73 upregulation in tumor-associated macrophages, and then suppressing the anti-tumor immune response, which provides a critical metabolic immune checkpoint in the senescent tumor microenvironment (55).

### 3.4 Express “Don’t eat me” signals

Evidence indicates that senescent cells inhibit the phagocytic function of macrophages by upregulating the expression of CD47, thereby conveying the “do not eat me” signals that originate from the inhibitory receptor signal-regulatory protein alpha(SIRPα), thereby suppressing cell engulfment (56). Daniela Schloesser et al. discovered that senescent cells were neither killed nor engulfed by macrophages by using two-cell *in vitro* co-cultures and demonstrated that senescent cells are refractory to macrophage engulfment or killing, which is closely related to the above-mentioned mechanism (57). This process, known as senescent cell engulfment suppression (SCES), is independent of the senescence-associated secretory phenotype (SASP) but instead requires direct contact between macrophages and senescent cells, and involves enhanced CD47 expression on senescent cells, coinciding with increased levels of the CD47-modifying enzymes QPCT/L (58). CD47, which serves as a “ do not eat me “ signaling molecule, is used by healthy cells to avoid being mistakenly phagocytosed, whereas senescent cells and tumor cells overutilize this mechanism to evade immune clearance (59). In addition, CD24 also acts as a “ do not eat me “ signal molecule by interacting with the inhibitory receptor Siglec-10 on the surface of macrophages, thereby inhibiting the phagocytic activity of macrophages in tumor cells and helping tumor cells evade clearance by the immune system (60). However, there are relatively few studies on the direct relationship between CD24 and cellular senescence. Senescent cells may also contribute to immune cell dysfunction, thereby weakening anti-tumor immune responses, and CD24 may play a certain role in them. This mechanism requires further investigation.

The preceding discussion elucidates the dualistic role of cellular senescence in tumor immunity: it can serve as a target for immune-mediated clearance, yet it may also function as a catalyst for immune escape. This fundamental paradox compels us to ask: what decisive factors govern the fate of senescent cells, flipping the switch from tumor “suppressor” to “accomplice”? Here, we move beyond a descriptive catalog of opposing outcomes to dissect the integrated determinants of fate that govern whether the senescence program ultimately serves as a guardian or an accomplice of cancer. We propose a multi-layered framework encompassing intrinsic cellular programs, extrinsic microenvironmental cues, and decisive spatiotemporal dynamics. Understanding these determinants is essential not only for deciphering the biological complexity but, more importantly, for actively intervening in this process to harness the pathological potential of senescence for therapeutic benefit.

## 4 Balancing the scales: what determines the fate of SCs

The decisive factors governing the fate of SCs can be conceptualized across three interconnected layers: the gene and epigenetic regulatory layer, the cytokine and chemokine secretome layer, and the dynamic microenvironmental interaction layer. The integrated output of these three tiers ultimately determines whether senescent cells function as “guardians” against cancer or as “accomplices” in tumor progression and immune evasion.

### 4.1 Cell-intrinsic programs

Acute DNA damage or oncogene-induced senescence, governed primarily by the p53/p21 axis, constitutes a rapid and potent cellular response to severe genomic threats (61). This form of senescence is characterized by a robust immunogenic profile. SCs actively secrete “find-me” signals, such as specific chemokines (eg. CXCL10, CCL5, CCL2), which recruit both innate and adaptive immune effectors to the site. Furthermore, they upregulate surface stress ligands, rendering themselves prominent targets for recognition and elimination by natural killer (NK) cells and CD8^+^ cytotoxic T cells. This process not only enhances immune recognition but also initiates adaptive immunity by supplying antigen-presenting cells with a milieu rich in antigens and pro-inflammatory signals (62).Ideally, this cascade triggers a potent immune surveillance and clearance program capable of effectively eradicating pre-malignant cells. Consequently, inducing this immunogenic senescent state represents a key therapeutic goal, as it harnesses the body’s own immune system for tumor suppression. Due to its strong immunogenic properties, p53/p21-driven senescence is typically more susceptible to rapid immune-mediated recognition and clearance.

In contrast, senescence driven primarily by the p16/Rb pathway typically arises in response to persistent low-level stress, such as telomere attrition or chronic oxidative damage (63). This form of senescence is characterized by a “pro-fibrotic and immunomodulatory” SASP, featuring factors like TGF-β, CTGF, PAI-1, and PGE_2_. This secretory profile drives stromal fibrosis and angiogenesis, thereby remodeling the physical architecture of the tissue microenvironment (64). Con-currently, the secretion of these and other inhibitory factors actively suppresses effector immune responses and fosters a state of immune tolerance. While maintaining a chronic, low-grade inflammatory tone, this process ultimately disrupts tissue homeostasis (65). Due to its weak intrinsic immunogenicity, this senescent phenotype often evades rapid immune clearance, leading to the persistent accumulation of senescent cells within tissues. This persistence is pathologically significant: by reshaping the microenvironment, these cells create protective niches for tumor cells, actively suppress anti-tumor immunity, and promote immune escape. Consequently, p16/Rb-driven senescence is closely associated with tissue aging, therapy resistance, and tumor progression (66).

These two senescence subtypes are not rigidly distinct but represent points on a dynamic continuum, with considerable overlap and plasticity in their phenotypes. For instance, an acute DNA damage response that is not fully resolved can transition into a state of chronic cellular stress. Furthermore, sustained activation of the p53 pathway may ultimately lead to the upregulation of p16, illustrating the mechanistic interplay between these regulatory axes (67).

### 4.2 The heterogeneity of the SASP and dynamic micro-environment interaction

As established in prior sections, the composition of the SASP serves as the direct molecular determinant that enables SCs to exert opposing immunomodulatory effects. Critically, this heterogeneity is not stochastic but is governed by defined upstream regulatory signals (62, 65). Moreover, cells from different tissue sources (such as fibroblasts, epithelial cells, and immune cells) may have very different SASP profiles even after experiencing the same stress. For example, senescent fibroblasts frequently upregulate factors involved in extracellular matrix remodeling and inflammation, such as MMP-1, MMP-3, and CXCL1. In contrast, senescent epithelial cells often shift their secretion toward angiogenic and growth-promoting factors like VEGF (68). This cell-type-specific divergence is strikingly illustrated by a direct proteomic comparison of senescent lung fibroblasts and renal epithelial cells, which revealed minimal overlap in their significantly upregulated secretomes; the vast majority of secreted factors were unique to each cell type (69).

Within tumor tissues, multiple senescent cell types—including tumor cells, cancer-associated fibroblasts (CAFs), and immune cells—coexist, collectively forming a dynamic “SASP ecosystem”. This ecosystem generates competing signals that critically shape the local immune milieu (70). For instance, senescent CAFs typically secrete factors that promote fibrosis and immunosuppression, thereby providing survival signals for tumor cells and inhibiting immune effector function. In contrast, coexisting senescent tumor cells may release immunostimulatory chemokines that recruit effector T cells (71). The ultimate immunological outcome within a given tumor region is determined by the net balance of these opposing secretory signals. During early disease stages or following certain therapies, immunostimulatory SASP signals from senescent tumor cells may predominate, tipping the balance toward effective anti-tumor immunity. However, in advanced or chronic settings, the persistent, dominant secretion of immunosuppressive and pro-fibrotic factors by senescent stromal cells (e.g., CAFs), coupled with a time-dependent shift in the SASP profile toward a more inflammatory and matrix-remodeling state, often overwhelms pro-immunogenic signals. This leads to the establishment of a suppressive microenvironment conducive to immune evasion and tumor progression (71).

It is precisely this spatially distributed interplay among different senescent cell populations and their competing secretory outputs that underlies the remarkable spatial heterogeneity of immune cell infiltration and activity observed within individual tumors. Deciphering the operational logic of this senescent cell ecosystem is therefore paramount for developing precise therapeutic strategies aimed at selectively eliminating harmful senescent subsets or reprogramming their SASP toward an anti-tumor phenotype (5).

## 5 Potential therapeutic strategy

The framework outlined above highlights a central therapeutic dilemma: senescence acts as a profoundly context-dependent double-edged sword within tumor immunity. Consequently, any monolithic therapeutic approach—be it the broad induction, blanket inhibition, or non-selective elimination of senescent cells—risks limited efficacy or even adverse outcomes due to a lack of precision. To address this, we propose a rationally staged interventional strategy: first, to induce senescence under controlled conditions, and subsequently, to selectively eliminate the senescent subpopulations that exert net harmful effects, timed to maximize therapeutic synergy and minimize pro-tumorigenic consequences.

### 5.1 Inducing “beneficial” senescence

Therapeutic induction of cellular senescence represents a viable intervention strategy to constrain tumor progression, particularly when applied during the early stages of disease development (72). As previously indicated, not all forms of senescence are immunosuppressive. Through targeted therapeutic intervention, tumor cells can be directed to enter a specific immunostimulatory senescent state. Cells in this state, while experiencing cycle stagnation, secrete specific cytokines and chemokines. This enables the recruitment and activation of the immune system, transforming immune “cold” tumors into immune “hot” ones, and creating favorable conditions for subsequent immunotherapy (73). Combining senescence-inducing therapies with the mobilization of the immune system represents a highly promising strategy, a premise that has been strongly substantiated by preclinical models. In mouse models of pancreatic ductal adenocarcinoma (PDAC), combined mitogen-activated protein kinase (MAPK) kinase and cyclin-dependent kinase 4/6 (CDK4/6) inhibitors have been shown to induce RB protein-mediated senescence program in tumor cells. This therapy-induced senescent state is accompanied by the production of SASP, which in turn acts to recruit and activate CD8^+^ T cells, thereby driving their accumulation into tumors that are otherwise immunologically “cold” (74). In models of KRAS-mutant lung cancer, combined inhibition of the MAPK and CDK4/6 pathways has also been to exert a dual antitumor effect. This regimen not only directly suppresses cancer cell proliferation but also engages the innate immune system by provoking NK cell-mediated surveillance program that contributes to tumor cell death. Mechanistically, this therapeutic combination promotes RB protein-mediated cellular senescence and activates a characteristic immunomodulatory SASP. Within this SASP, specific factors—notably tumor necrosis factor-α (TNF-α) and intercellular adhesion molecule-1 (ICAM-1)—have been identified as critical mediators required for the efficient recruitment, recognition, and elimination of drug-treated tumor cells by NK cells (75).

While inducing immunogenic senescence serves as a promising therapeutic “ignition” strategy, its efficacy can be constrained by tumor microenvironment heterogeneity and the dynamic phenotypic plasticity of senescent cells themselves. Not all therapy-induced senescent cells sustain a pro-immunogenic state; some may evade timely clearance or, under chronic inflammatory pressure, undergo a secretory shift from immune-stimulatory to immunosuppressive, effectively turning from allies into threats (76). Therefore, following successful immune activation, a critical parallel objective emerges: to identify and eliminate pre-existing or therapy-generated senescent cell populations that exhibit pro-tumorigenic potential. This refocuses the therapeutic approach from induction to targeted clearance—the strategic application of senolytic agents.

### 5.2 Eliminating “harmful” senescence:application of senolytics

Senolytics are drugs that selectively eliminate senescent cells and have been widely studied for cancer treatment in recent years, such as BH3 mimetics, flavonoids (e.g. quercetin, fisetin), FOXO4 peptidomimetics, galacto-conjugated compounds, cardiac glycosides, as well as other small molecules, such as HSP90 chaperone inhibitors and bromodomain and extraterminal domain (BET) protein degraders (BETd) (77). A particularly promising application of this principle is the “one-two punch” therapeutic strategy. This sequential approach first induces senescence in cancer cells (the “first punch”), thereby creating a population of therapy-induced senescent cells, which are then selectively eradicated by a senolytic agent (the “second punch”). For instance, Yingjie Qing et al. used wogonin to induce cellular senescence in T-cell malignancies at a nonlethal concentration, whose mechanisms involved inhibition of telomerase activity, DNA damage, as well as upregulated expression of BCL-2, and then identified a BCL-2 inhibitor, Navitoclax (ABT-263), which can decrease cell viability and induce apoptotic cell death in wogonin-induced senescent cells, thereby demonstrating that the “one-two punch” approach can increase the sensitivity of T-cell malignancies with low BCL-2 expression to Navitoclax (78).

### 5.3 Synergistic immunotherapy

By selectively eliminating pro-tumorigenic senescent cells, senolytic agents directly deplete a major source of immunosuppression within the tumor microenvironment. The therapeutic impact, however, extends beyond mere cellular depletion. This clearance actively remodels the immunological landscape by attenuating inhibitory signals, potentially enhancing T-cell infiltration, and releasing tumor antigens. Together, these effects establish a critical therapeutic window—a temporally defined microenvironment in which immune suppression is alleviated and effector cell function may be restored. Therefore, a logical therapeutic extension involves combining senolytic clearance with immunotherapies designed to reactivate T cells, such as immune checkpoint inhibitors (ICIs) (79). This integrated strategy is postulated to yield a synergistic effect: senolytics act to “remove the roadblocks”, while immunotherapy is responsible for “accelerating the attack”, jointly overcoming the immune escape mechanism of tumors.

In colorectal cancer models, the combined administration of PARP inhibitors and CDK4/6 inhibitors induces a therapy-induced senescence program that is critically dependent on the cGAS/STING signaling pathway. This specific senescent phenotype plays a pivotal role by reprogramming the tumor immune microenvironment, effectively converting tumors from a non-responsive, “immune-cold” state into one that is highly sensitive to PD-L1 blockade. Consequently, this sequential approach generates a marked synergistic anti-tumor effect (80). Ashkan Shahbandi et al. further demonstrated that chemotherapy-induced senescence activates distinct immune-modulatory programs, notably upregulating the PD-1/PD-L1 axis to mediate T-cell inhibition. Importantly, this senescence-associated PD-L1 expression predicts favorable responses to subsequent immune checkpoint blockade. Based on this, they proposed a “prime-and-block” strategy: chemotherapy serves to “prime” the tumor by inducing senescence and exposing PD-L1, after which a PD-L1 inhibitor “blocks” this immune checkpoint to prevent evasion and enhance immune-mediated clearance of senescent cells. This work positions PD-L1 as a predictive biomarker for identifying patients most likely to benefit from combined chemo-immunotherapy (81).

Persistent SCs post-chemotherapy act as a reservoir for tumor relapse and limit tumor clearance after chemotherapy, partly due to PD-L2-mediated immunosuppression, PD-L2 exerts a stronger inhibitory effect on T cells compared with PD-L1, recruiting MDSCs to establish an immunosuppressive microenvironment, and directly protecting senescent cells from being cleared by CD8+T cells (82). Selim Chaib et al. demonstrated that combining chemotherapy with anti-PD-L2 therapy enhances tumor clearance compared to chemotherapy alone in preclinical models. Their work systematically established that PD-L2 is robustly upregulated following chemotherapy-induced senescence across multiple malignancies, including head and neck carcinoma, endometrial cancer, and melanoma. This positions PD-L2 not only as a predictive biomarker of therapy-induced senescence but also as a compelling therapeutic target whose blockade can augment the efficacy of standard chemotherapy (53).

Chimeric antigen receptor (CAR) T-cell therapy represents a groundbreaking advancement in tumor immunotherapy, through genetic engineering, T cells can be modified to express a CAR that specifically recognizes tumor-associated surface antigens, thereby reactivating cytotoxic T-cell responses against malignant cells (83). Senescent tumor cells can express specific surface proteins, such as urokinase plasminogen activator (uPAR). Amor et al. demonstrated that uPAR is a cell-surface protein that is highly expressed during senescence by comparing RNAseq datasets derived from models of senescence. Furthermore, uPAR-specific CAR-T cells effectively eliminated SCs both *in vitro* and *in vivo* by recognizing the surface antigens of SCs. This approach directly kills SCs via the perforin/granzyme pathway and simultaneously inhibits the pro-inflammatory and pro-fibrotic effects of SASP (such as reducing the secretion of IL-6 and TGF-β), prolonging the survival of mice with lung adenocarcinoma treated with senescence-inducing drugs (84).

Although the logic of synergy is solid and the preclinical data is encouraging, this strategy still faces key challenges in moving towards clinical practice. Future clinical trial designs need to be particularly meticulous to verify whether this smart therapy that combines “breaking barriers” and “accelerating” can bring lasting benefits to more patients.

## 6 Future directions and challenges

The interplay between cellular senescence and tumor immunity represents a double-edged sword. While senescence acts as a potent tumor-suppressive barrier through cell-cycle arrest, immunogenicity, and the anti-tumorigenic effects of SASP, its chronic persistence fosters an immunosuppressive milieu that ultimately aids tumor immune escape. We need precision tools to selectively inhibit detrimental factors while preserving beneficial ones. Notably, tumor cell senescence induction exerts off-target effects on adjacent healthy tissues. A therapeutic strategy that activates the senescence pathways of tumor cells represents a promising avenue for future anticancer discoveries. In addition, non-specific clearance of senescent tumor cells may inadvertently damage normal senescent cells (e.g., hepatic stellate cells causing fibrosis, or vascular endothelial cells exacerbating atherosclerosis, etc.), highlighting the urgent need for tumor-restricted senotherapeutics. Emerging solutions like antibody-drug conjugates targeting senescence-specific surface markers (e.g., uPAR) show promise, yet their clinical translation remains constrained by unresolved pharmacokinetic challenges, including rapid clearance and target-mediated drug disposition. These challenges, while daunting, represent measurable gaps rather than fundamental limitations—a roadmap for the next decade of translation.
